# A Case of Double Empyema

**Published:** 1893-12

**Authors:** William Johnstone Fyffe


### A CASE OF DOUBLE EMPYEMA.

BY
WILLIAM JOHNSTONE FYFFE, B.A., M.D. Dub.

CASES of bilateral pleurisy followed by empyema are not of very frequent occurrence. I have found records of a few cases, but they have been principally among persons of from 40 to 60 years of age, and some of them appear to have been of pyæmic or tubercular origin. Fraentzel, in his article on "Diseases of the Pleura" in Ziemssen's *Cyclopædia*, states that the extension of the inflammation from the pleura primarily attacked to the unaffected side is as a rule only observed when the former is the seat of tuberculosis. Hence, he says, we may establish with tolerable certainty the diagnostic fact, which is based on experience, that when pleuritis appears simultaneously on both sides, it is commonly of tubercular nature. As, however, tubercular pleuritis seldom appears as a primary affection, the extension of the disease from one pleura to the other in primary *non*-tubercular pleuritis is of very rare occurrence. It appears to me, therefore, that a case of non-tubercular double pleurisy occurring in a healthy boy, complicated with pneumonia of the left lung, followed by empyema on both sides of the chest, and ending in complete recovery after evacuating the abscesses by operation and drainage, is one of sufficient interest to place on record.

H. S., aged 17, a Clifton College boy and member of the South Town House, had an ordinary catarrhal cold about the 18th February last. On the 21st I was called to see him, and found him very flushed, breathing about 30 times in the minute, with a pulse of 100 and a temperature of 103°. On examination I found dulness on percussion at the base of the right lung. The dulness was marked but not absolute. There was coarse crepitation extending up to the angle of the scapula. There was pain on this side extending round to the front of the chest, but the respiratory sounds in the anterior part of the lung were healthy; he could lie on either side, but the *decubitus* was mainly on the left. I diagnosed the case as one of pleuro-pneumonia, and treated it by external applications, poulticing, and regular feeding with fluid food. Except to ensure free action of the bowels, very little medicine, except an occasional dose of phenacetin, was given.

The symptoms during the first week were not of a very acute character, but at the end of that week the patient became more oppressed, and it was evident that no resolution of the inflammation was taking place. The left lung at this time remained intact. He continued much in the same condition till about the 4th March, when I found that the inflammation had attacked the other side of the chest. There was severe pain in the left side and back, and distinct friction-sound. There was dulness on percussion over a space of a hand's-breadth at this point, and immediately above this and close to the angle of the left scapula there was a distinct patch of pneumonia, circumscribed, and occupying the centre of the posterior part of the left lung. In the right lung, which was first attacked, the signs of inflammation of the pleura were less severe, but at the same time undoubted. There was no displacement of the heart. The general symptoms, however, had become aggravated, and the pulse-rate and number of respirations had increased.

At the end of the third week there was a decided increase in all the physical signs, and there was no question that fluid of some kind was present in both pleuræ; and the time had come when some definite action must be taken for the patient's relief. Moreover, an irritative and constant cough had set in, accompanied with purulent expectoration, and there were severe night perspirations and great emaciation. There was evidently a communication between the empyema and the right bronchus.

On March 13th, after consultation with Dr. E. Long Fox and Dr. James Swain, who had taken charge of the case during my absence for a week, it was decided to make an exploratory puncture in the left side in the ninth interspace. This was done at once by Dr. Swain, and the syringe was filled with a sero-purulent fluid. We therefore concluded to open the chest the same day at the point where the puncture had been made. Chloroform was most carefully administered by Mr. Dacre, and the chest was freely incised by Dr. Swain in the ninth interspace. The patient bore the anæsthetic very well, and there was no subsequent sickness.

Dr. James Swain has kindly supplied me with the following notes:—

"March 13th. It was thought best to open the left side of the chest, which was universally deficient in resonance. A small area below and to the inside of the angle of the left scapula was found to be absolutely dull. After the administration of a small amount of chloroform an incision was made at the ninth intercostal space, about three inches to the left of the spinal column. Pus was evacuated from a cavity about as large as the closed fist, and surrounded by adhesions of recent lymph, glueing the visceral and parietal layers of the pleura together. An empyema tube was placed in the wound, covered with a valve of gutta-percha tissue and dressed with wood-wool wadding.

"On the 15th the abscess in the right pleural cavity was opened in a similar manner. From this cavity there escaped about 10 or 12 ounces of pus, and the tube was placed in the eighth intercostal space, just below and in front of the angle of the right scapula.

"The cavity on the right side became septic owing to the communication which, previous to the operation, had taken place with the right bronchus; but the slight elevation of temperature caused by this soon ceased after leaving about a drachm of boro-glyceride solution in the cavity when dressed.

"At first the discharge from both tubes was very profuse, but it gradually lessened, and at the end of four weeks the left tube was taken out; and the right was taken out about five and a-half weeks after the operation. The patient, who at first was so weak that he could be scarcely turned in bed, made an uninterrupted recovery, aided by large quantities of food and stimulants. He was able to get to a couch about three and a-half weeks after the operations; and on May 1st, six weeks after the operations, he was sent to Weston-super-Mare to complete his convalescence.

On this date I made the following note: Impaired percussion resonance over both sides of the back. Vocal resonance and fremitus about normal. Vesicular breathing on both sides down to the bases. The cyrtometer shows an equality of expansion on both sides of the chest. The track of the right tube is not quite healed, but that of the left is practically well.

"There are a few points of interest to which it is desirable to refer. Speaking of the case generally, it may be said that the successful issue was very largely due to the great amount of plastic exudation, which had bound down both lungs to such an extent that the collapse which necessarily ensues when an opening is made into the pleural cavity was markedly limited, and the respiratory capacity thereby proportionately benefited.

"All observers are agreed that in cases of double empyema it is desirable to allow a week or more to elapse between the times of operation on each side of the chest; but in this case we did not feel justified in waiting more than 48 hours, and we knew that the lung on the left side, which was opened first, was very largely adherent to the parietes.

"The case also exemplifies how small is the pathological difference between the so-called localised and the general empyema. Although practically the local empyema may be regarded as the less serious, it would appear as if the amount of localisation or generalisation was simply a question of greater or less amount of agglutination between the two layers of the pleura.

"With reference to the tube employed, I used the ordinary flanged tube, tied round the chest with tape attached to either flange. The tube was covered with a piece of gutta-percha tissue so arranged over the lumen that when wet it acted as a valve, permitting only of expiration through the tube, and during inspiration fitting so closely over the end of the tube that practically no air entered the pleural cavity. Of this simple method of treating the empyema tube I can speak very favourably. In this case vesicular breathing could be heard over the whole chest in the course of three or four days, except in the immediate vicinity of the tubes, showing how closely the lung had come in contact with the chest wall, being aided of course by the yielding of the chest wall itself to the external pressure.

A potential cavity, however, still existed between the two layers of the pleura, which were not yet adherent, and in finally withdrawing the tubes we were guided by the cessation of purulent discharge rather than by auscultatory physical signs."

We all know how satisfactory as a rule cases of single empyema occurring in young people are, when we have at our hand all the advantages which antiseptic surgery bestows. I am old enough to recall a very different experience, and think with regret on the number of lives I have seen lost which might have been saved if only the surgical knowledge which now seems so simple had been available.

But a double empyema with a patch of pneumonic consolidation in one lung was a condition of things I had not met with before, and I confess that both before and after the operations were performed I had the gravest fears for the result; and I should be doing a great injustice to my surgical colleague, Dr. James Swain, if I did not say emphatically that the successful issue to which this case has been brought has been mainly owing to his skill, care, and continuous attention. Material assistance was also rendered by the admirable and efficient nursing the patient received from his sister.

The patient's condition at present is a satisfactory one. There is good respiration over both lungs, and I have every reason to hope that no permanent damage has been done. He has recovered strength, weight, and colour; his voice is strong; and he has returned to athletics and class work.

---

